# A method for quantitative evaluations of scanning‐proton dose distributions

**DOI:** 10.1002/acm2.13226

**Published:** 2021-03-29

**Authors:** Bryce C. Allred, Jie Shan, Daniel G. Robertson, Todd A. DeWees, Jiajian Shen, Wei Liu, Joshua B. Stoker

**Affiliations:** ^1^ Department of Radiation Oncology Mayo Clinic Arizona Phoenix AZ USA; ^2^ Department of Health Sciences Research Mayo Clinic Arizona Phoenix AZ USA

**Keywords:** PSQA, gamma test, proton

## Abstract

**Purpose:**

Patient‐Specific Quality Assurance (PSQA) measurement analysis depends on generating metrics representative of calculation and measurement agreement. Considering the heightened capability of discrete spot scanning protons to modulate individual dose voxels, a dose plane comparison approach that maintained all of the capabilities of the well‐established γ test, but that also provided a more intuitive error parameterization, was desired.

**Methods:**

Analysis was performed for 300 dose planes compared by searching all calculated points within a fixed radius around each measured pixel to determine the dose deviation. Dose plane agreement is reported as the dose difference minimum (DDM) within an empirically established search radius: ΔDmin(r). This per‐pixel metric is aggregated into a histogram binned by dose deviation. Search‐radius criteria were based on a weighted‐beamlet 3σ spatial deviation from imaging isocenter. Equipment setup error was mitigated during analysis using tracked image registration, ensuring beamlet deviations to be the dominant source of spatial error. The percentage of comparison points with <3% dose difference determined pass rate.

**Results:**

The mean beamlet radial deviation was 0.38mm from x‐ray isocenter, with a standard deviation of 0.19mm, such that 99.9% of relevant pencil beams were within 1 mm of nominal. The dose‐plane comparison data showed no change in passing rate between a 3%/1mm ΔDmin(r) analysis (97.6 +/‐ 3.6%) and a 3%/2mm γ test (97.7 +/‐ 3.2%).

**Conclusions:**

PSQA dose‐comparison agreements corresponding to a search radius outside of machine performance limits are likely false positives. However, the elliptical shape of the γ test is too dose‐restrictive with a spatial‐error threshold set at 1 mm. This work introduces a cylindrical search shape, proposed herein as more relevant to plan quality, as part of the new DDM planar‐dose comparison algorithm. DDM accepts all pixels within a given dose threshold inside the search radius, and carries forward plan‐quality metrics in a straightforward manner for evaluation.

## 1 INTRODUCTION

Patient‐Specific Quality Assurance (PSQA) is a common practice for intensity modulated radiation therapy with proton (IMPT) and x rays (IMRT) in order to verify agreement between the calculated and delivered doses. The measurement methods have evolved in step with the proliferation and increasing complexity of IMRT: contemporarily, measurement of the planned delivery is performed on a planar or cylindrical array, and then cross compared with a treatment planning system (TPS) calculation, with the level of agreement being described by a γ index (see Eq. 1) as proposed by Low et al.^1^

There are two parameters which define the γ index: Distance to Agreement (DTA or Δd_M_) and Dose Difference (ΔD_M_). These criteria define the passable agreement tolerances. The DTA is classically defined as a distance between a measured point and the nearest calculated pixel with a dose value that agrees within a set threshold.^2^ The DTA commonly used for the γ index is conflated with a dose agreement criterion to define a passing (γ < 1) ellipse; this search shape results in the dose threshold decreasing as the DTA increases. The spatial axis of the ellipse is defined by taking the three‐dimensional (3D) distance between the locations of the measured point within the delivered dose volume and a test point in the TPS dose volume, then dividing it by the DTA(Δd_M_). The dose axis of the ellipse is defined by the difference in dose between the measurement and the same test point from the TPS volume, divided by the local or global maximum dose from the plane, and then divided by ΔD_M_. For each measured point, there are many (N) tested points within the TPS volume depending on the search grid resolution.

(1) $$\gamma ‐{\text{index}}_{n}=\min\left\{\sqrt{{\left(\frac{\Delta {\text{Dist}}_{n}^{\text{meas}‐\text{test}}}{d_{M}}\right)}^{2}+{\left(\frac{\left(\frac{\Delta {\text{Dose}}_{\text{meas}‐\text{TPS}}^{n}}{\text{Max Dose}}\right)}{D_{M}}\right)}^{2}}\forall n\in \left\{1:N\right\}\right\}$$

A resulting γ index <1 indicates that the measured point has passed the correlation test. TG 119 recommended a 90% passing rate for a Δd_M_ = 3mm and ΔD_M_ = 3%, considering >10% relative dose ^3^, though PSQA was a secondary consideration of the report. The more recent PSQA‐dedicated TG 218^4^ suggested a passing rate tolerance of 95%, and a passing rate action level of 90%, assuming test thresholds of ΔD_M_ = 3% and Δd_M_ = 2mm for pixels receiving >10% of intended dose.

The γ test gained widespread acceptance relatively soon after being introduced; test results provide a simple summary of the somewhat complex interplay between beam alignment and dose accuracy. The now widespread availability of intensity modulated beam delivery, including IMRT and volumetric moldulated arc therapy (VMAT), spurred the demand for increasingly sophisticated PSQA diagnostics.^5^, ^6^, ^7^, ^8^, ^9^ Specifically, previous work has denoted γ‐test shortcomings in several key parameters: spatial sensitivity,^6^, ^9^, ^10^, ^11^, ^12^ dose sensitivity^5^, ^8^, ^10^, ^13^, ^14^, ^15^ and specificity.^7^, ^16^, ^17^ While insightful and formative to this work, these reports did not address some challenges unique to evaluating IMPT dose distributions.

In an effort to test the sensitivity of our institution’s PSQA process for IMPT delivery, dose deviations of up to 15% were inserted into a 3x3cm^2^ plane within a 10x10x10 cm^3^ homogenous dose cube. The γ test was employed to compare the various deviated deliveries to the original treatment plan. It was determined that a 5% dose deviation could yield a γ‐passing rate identical to a significantly higher dose deviation of similar area. TG 218 recommends that in addition to passing rate, users also review both maximum γ values, as well as the percentage of γ values >1.5. These additional metrics provide users a way to monitor for extreme delivery deviations, but the test’s intrinsic conflation of dose and DTA (see Eq. 1) makes it impossible to isolate either parameter as the source of the error. Both parameters are currently computed as an intermediate result of the traditional elliptical γ test. However, the division step in Eq. 1 ultimately renders both the distance and dose parameters unitless prior to the final‐output index value. We determined that minor adjustments to the γ test could carry both distance and dose deviation per pixel forward to the end user for evaluation. Since each spot is delivered indepently during discrete spot scanning delivery, these systems exhibit a heightened capability to modulate individual dose voxels relative to IMRT. Consequently, a plane‐comparison approach that maintained all of the capabilities of the well‐established γ test, but that also provided a more intuitive error parameterization, was desired.

Although the elliptical bound has evolved today into widespread use among PSQA applications, a\ cylindrically bounded dose agreement search space was also suggested by Low et al.^1^ A cylindrically bounded search shape operates such that the dose agreement level is invariant over the full search space, as opposed to the generally applied elliptical γ test that applies a dynamic dose tolerance that decreases as DTA increases. This complimentary concept is explored in this work as a means of: 1) emphasizing spatial accuracy; 2) allowing the user to quantify dose deviation magnitude and direction; 3) maintaining an invariant dose tolerance over the entire search space. These aims are accomplished via output of a dose deviation histogram as a function of search distance.

The proposed function receives the same inputs as the familiar γ test, such that no modification to current two‐dimensional (2D) array data acquisition workflows are required. However, this approach replaces DTA by a fixed search distance, *r*, and reports the dose deviation minimum per pixel, ΔD_min_(*r*), or DDM, within the area (or volume for 3D analysis) bounded by *r*.

Fixed spatial tolerances are based on in‐depth characterization of our proton radiation delivery system that, along with TG‐224 standards,^18^ indicate that modern accelerators are capable of submillimeter spatial accuracy. Defining the search distance based on measured accelerator performance enabled a dose analysis considered by this work to be more relevant to defining the quality of beam delivery. In contrast, other approaches to planar dose comparisons with search regions much larger than anticipated radiation beamlet accuracy, including the γ test, allow for false positives that inflate quality metrics.

The linear relationship of γ criteria and action levels assessed by Crowe et al,^7^ along with the assertion that “no [γ] threshold will provide both high sensitivity and high specificity”^16^ suggests that something other than an elliptical passing volume is necessary in order to optimize sensitivity and specificity. This manuscript demonstrates an approach sufficiently sensitive to determine error in highly modulated radiation therapy as well as sufficiently robust that non‐clinically relevant errors do not cause failure in all cases examined.

## 2 METHODS AND MATERIALS

### 2.1 Experimental Setup

PSQA measurements for proton fields were performed using the Matrixx PT ion chamber array inside of a Digiphant water phantom (IBA Dosimetry, Schwarzenbruck, Germany). Measurements were taken with a constant gantry angle (90°) and fixed source to surface distance (isocenter at depth of 10 cm). For each patient field, three planes were measured with the 7.62‐mm spacing of the Matrixx ion chambers, one proximal to the Spread Out Bragg Peak (SOBP), one within the prescription region of the SOBP, and one at the distal falloff of the SOBP.

A spot‐scanning proton beam provided the opportunity to directly measure parameters, such as beamlet position, from empirical measurements of the delivery system. Beamlet position accuracy was continuously monitored as part of our synchrotron delivery system. The synchrotron safety system tracks systematic and random beamlet position deviation to ensure the centroid and tail of the beamlet superposition remain within tolerance.

### 2.2 Search distance vs distance to agreement

In order to combine the dose and distance dimensions into a γ index, units are removed through division, thereby concealing the responsible parameter (dose or DTA), such that the user must back calculate via the γ angle and index values to tease out the per‐pixel dose or DTA. To achieve a fixed dose threshold across the search shape and a more straightforward ouptut, we elected to report per‐pixel dose deviations directly as a histogram for a given search distance threshold.

To support the proposed DDM method, a new parameter, named the Search Distance (*r*), is necessary. Unlike DTA, which is a variable in the γ index equation, *r* is fixed value defined per clinic from empirical determination of the spatial accuracy of the modulated beamlet. *r* defines a radius that outlines a circular area (or spherical volume) centered on each measured point, within which all calculated test points are compared to the measured point, ultimately recording the dose of the comparison point demonstrating the minimum dose deviation from the measured point. In contrast, a γ test may search outside of the set distance threshold if no nearby dose points fall within threshold, and report the corresponding DTA value, even though it may be larger than the threshold value. DDM does not search outside the set distance threshold, but instead relies on an empirical determination of a statistically relevant *r*. At the outset of this work, the authors sought to determine an *r* that would search over at least a 3σ confidence interval of beamlet accuracy (99.7%).

Alignment, or registration, of the fields negated the error components associated with setup and couch uncertainties, ensuring that the positions of the individual pencil beams were the dominant contributor to spatial uncertainty of the PSQA system. Relative plane shifts required during alignment were tracked to ensure that all adjustments fell within error tolerances of setting equipment up to lasers, and were consistent in magnitude and direction for each data collection session. Laser and x‐ray isocenter coincidence, and beam and x‐ray isocenter coincidence were checked daily prior to clinical use per institutional policy.

### 2.3 Dose difference minimum vs dose difference

The γ test uses a dose difference to define one axis of the elliptical space which defines acceptable agreement. In contrast, the proposed method reports a spatially limited Dose Difference Minimum: ΔD_min_(*r*) or DDM,

(2) $${\text{DDM}}_{n}=\min\left\{100*\left(\frac{\Delta D_{n}^{\text{meas}‐\text{TPS}}}{\text{Max Dose}}\right)\forall n\in \left\{1:N\right\}\right\}$$

where “*N*” is the number of test points within the area (volume) bounded by the empirically determined *r*, “$\Delta D_{n}^{\text{meas}‐\text{TPS}}$” is the difference in dose found between the measurement and the calculation from the TPS for the *n* ^th^ test point, and “Max Dose” is the global maximum of the dose plane. Similar to the available γ analysis algorithms, this algorithm correlates each measurement to a matched location within the calculated dose volume and then compares with multiple (*N*) dose points surrounding that location. The difference is that the dose threshold is invariant over the full search space. The minimum result from the *N* comparisons is still the accepted output, as with γ analysis, but now may be expressed directly as either absolute or relative dose for a given search distance. This function may be simply thought of as reporting the best dose agreement within a statistically probable search distance, per pixel.

### 2.4 Analysis

For comparative analysis with the DDM method, 300 dose planes from 75 IMPT patients previously planned at our clinic were selected; a 3%/2mm γ test, as well as a 3%/1mm DDM analysis, were performed for each. Plan selection was such that the full complement of treated disease sites, with treated volumes spanning the full field size capabilities, were included. The metrics for selection included: fields with abnormally large or abmormally small size (>18 × 18 cm^2^ or <4 × 4 cm^2^), 2%/2 mm γ pass rates below 95%, and disagreement in Max Dose between measured and calculated planes >5%. This was done in order to investigate whether specificity of the DDM technique was sufficient.

Additional γ analyses were run for 10 randomly selected IMPT patients with an in‐house modification to the γ script that recorded the DTA and dose difference values used to determine the γ index for each pixel, in order to track the percentage of per‐pixel DTA values that fell within 1.0 mm.

The Matrixx software suite was used to export the sampled beam as a planar array, scaled to Radiobiologically Equivalent units of cGy (RBE), for comparison with the corresponding TPS dose plane. An in‐house script was developed to automatically import, scale, and align the measured and calculated treatment dose arrays. The scaling was performed according to a normalization measurement taken prior to each measurement session to account for temporal output drift. The image registration algorithm only considered dose patterns above 20% of maximum dose for a coarse alignment with a Discrete Fourier Transform algorithm, then a least squares algorithm performed final shifts without the threshold. After the 2D arrays were aligned, the code compared each measured value, above a 10% of maximum dose threshold, to a grid of calculated test points within a defined search distance. No interpolation of the measured pixels was performed. The minimum dose variation from these comparisons was recorded into an array with the same dimensions as the measurement array. This resultant array was displayed as a heat map to indicate the magnitude and direction of the deviation for each pixel.

In order to determine the most appropriate parameters for designating agreement between calculated and measured dose planes, we implemented quadratic generalized linear models (GLM). Quadratic GLM allows for non‐linear associations and is strongly related to predictive modelling (i.e., *R* ^2^ yields proportion of variability explained and *r* depicts level of correlation). Spatial deviation for all measurements was characterized utilizing standard descriptive statistics (four moments) for location and variability. For completeness of statistical methodology, both parametric (Student’s T‐Test) and non‐parametric (Wilcoxon signed rank test) testing methods were utilized for analyzing the distribution of spatial deviation.

The data that support the findings of this study are available from the corresponding author upon reasonable request.

## 3 RESULTS

### 3.1 Search distance

Average beamlet position deviation from x‐ray isocenter was determined from high‐resolution (0.275 mm/pixel) scintillator measurements over a 6‐month period across all four treatment gantries.^19^ The resulting deviation histogram was weighted according to clinical use frequency, based on delivery logs collected over a 1‐month period, to determine the probability weighted distribution.

**Fig. 1:**
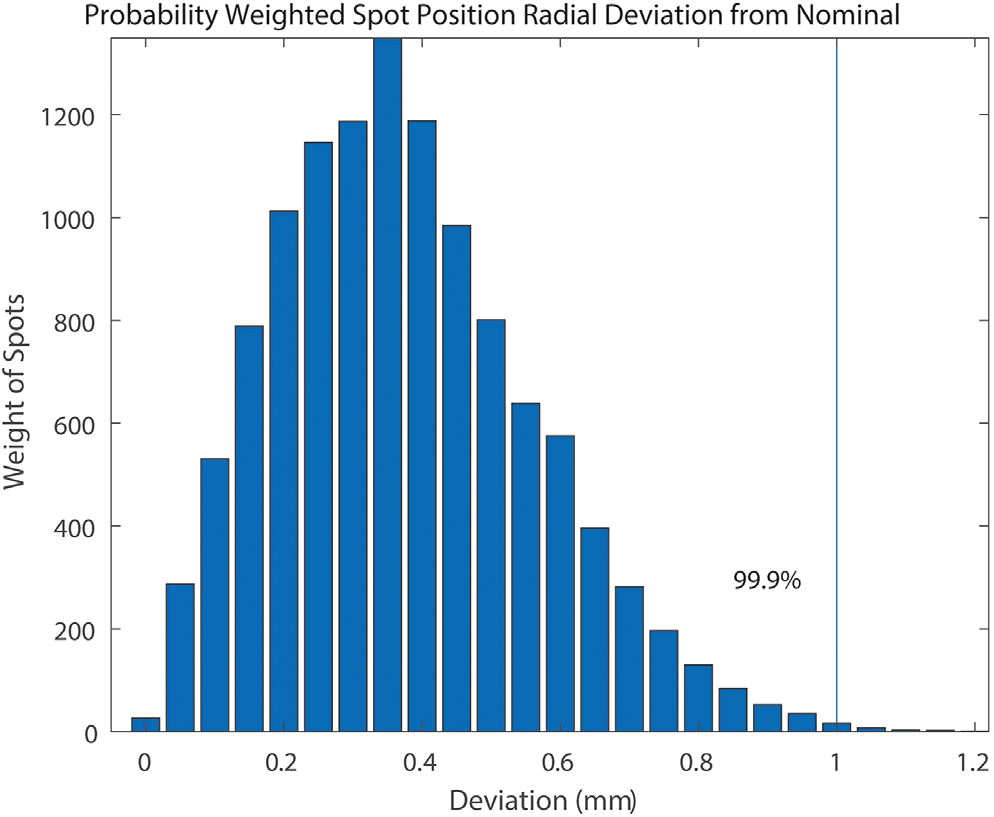
Probability‐weighted radial distance deviations of discrete proton spots from x‐ray isocenter. 99.9% of delivered spots probabilistically deviate from nominal by <1.0 mm. Data were measured over 30x40cm^2^ field size grid in four gantries over 6 months; probability density of spot deviations were determined based on all plans delivered over a 1‐month treatment period.

The mean beamlet radial deviation is 0.38mm from nominal, represented in Table 1, with a standard deviation of 0.19mm. It was determined that 99.9% of relevant pencil beams are delivered within 1 mm of the desired location (Fig. 1).

**Table 1:** Descriptive statistics & hypothesis tests for average beamlet deviation and the proportion of beamlet deviations <1 mm.

N	Range	Mean (SD)	1‐mm σ limit	% <1 mm	*P*‐value
11721	0.00‐1.6	0.38 (0.19)	3.26σ	99.9%	<0.0001*

* *P*‐values shown are for both Student’s t‐test and Wilcoxon signed rank test.

While the underlying distribution of deviations is non‐normal due to the restriction that deviations must be positive, large sample theory and the Central Limit Theorem allow for testing based on the normal distribution. Both the one sided Student’s t‐test and Wilcoxon signed rank test showed high statistical significance (*P* < 0.0001) for the probability that the average beamlet position deviation was less than 1 mm. Any agreement to test points beyond the determined range from the measured point is most likely coincidental, or a false positive, since beam position accuracy renders correlation statistically improbable (i.e., <0.1% for the observed distribution). Based on this characterization of our system, and in line with TG‐224 standard of 1‐ mm agreement between beam and x‐ray isocenter, an *r* = 1.0 mm was used ^18^.

### 3.2 Relative sensitivities: ΔD_min_ (r) vs γ index

**Fig. 2:**
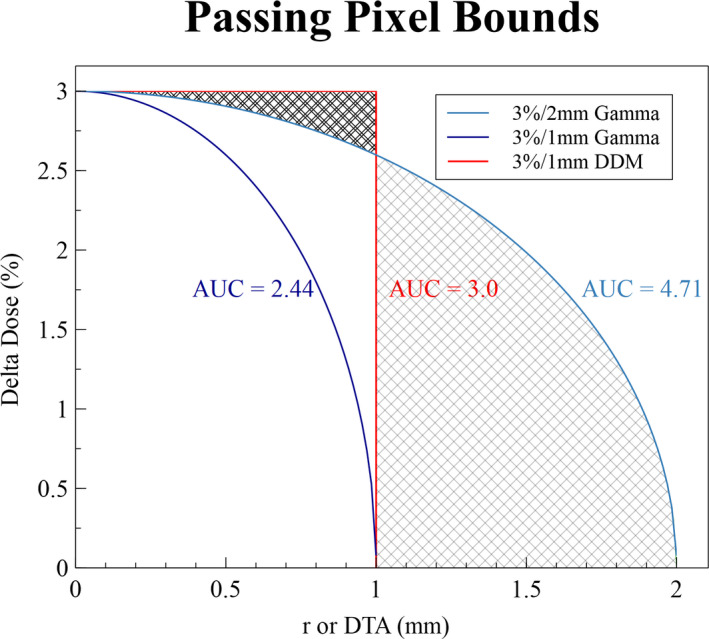
Area Under the Curve (AUC) displaying relative sensitivity of Distance to Agreement (DTA) and Dose Difference for two sets of γ index criteria: 3%/2mm AUC = 4.71 and 3%/1mm AUC = 2.36. The various γ sensitivities are represented by a single quadrant of each ellipse that vary in size. These are compared to the relative sensitivity of finding the dose difference minimum (DDM) within a fixed search distance: 3%/1mm AUC = 3.0, represented by a rectangle. The reader may readily observe that for a given distance threshold, the γ test is significantly more dose restrictive than the proposed DDM method, rendering use of the 3%/1mm γ test clinically intractable. The single‐hashed area represents distances outside of machine performance limits for our system, where γ agreements would have a high likelihood to be false positives; the double‐hashed area represents where the 3%/2mm γ is too dose restrictive, and reported failures would have a high likelihood to be false negatives.

We employed an area under the curve (AUC) analysis as a means to demonstrate the relative sensitivities of the ΔD_min_(*r*) and γ index approaches. As discussed in Jiang et al,^20^ an acceptance region can be delineated in two dimensions for AUC analysis by plotting the function for each test with dose on the vertical axis and distance on the horizontal axis (Fig. 2). The ellipse for the γ test is achieved by reflecting the curved line over the x‐axis and rotating about the y‐axis; the ΔD_min_(*r*) cylinder is visualized using the same reflection and rotation process. The relative dose and beam position sensitivities for the defined acceptance region can be correlated to the AUC for each line in Fig. 2. It is easy to observe that the greater the AUC, the more variation is allowed for passing a measurement. However, when characterizing the spatial accuracy of our proton accelerator we determined with 99.9% confidence that each beamlet would be delivered within 1 mm of the expected location. Therefore, we anticipate that dose congruence beyond 1‐mm radial deviation from intended location (indicated by single hash marks in Fig. 2) is highly indicative of a false positive, rather than true dosimetric agreement. To avoid these false positives, passing criteria should ideally be set based on the spatial tolerances of each machine, leading to test metrics better correlated to the quality of beam delivery.

### 3.3 Example output

**Fig. 3:**
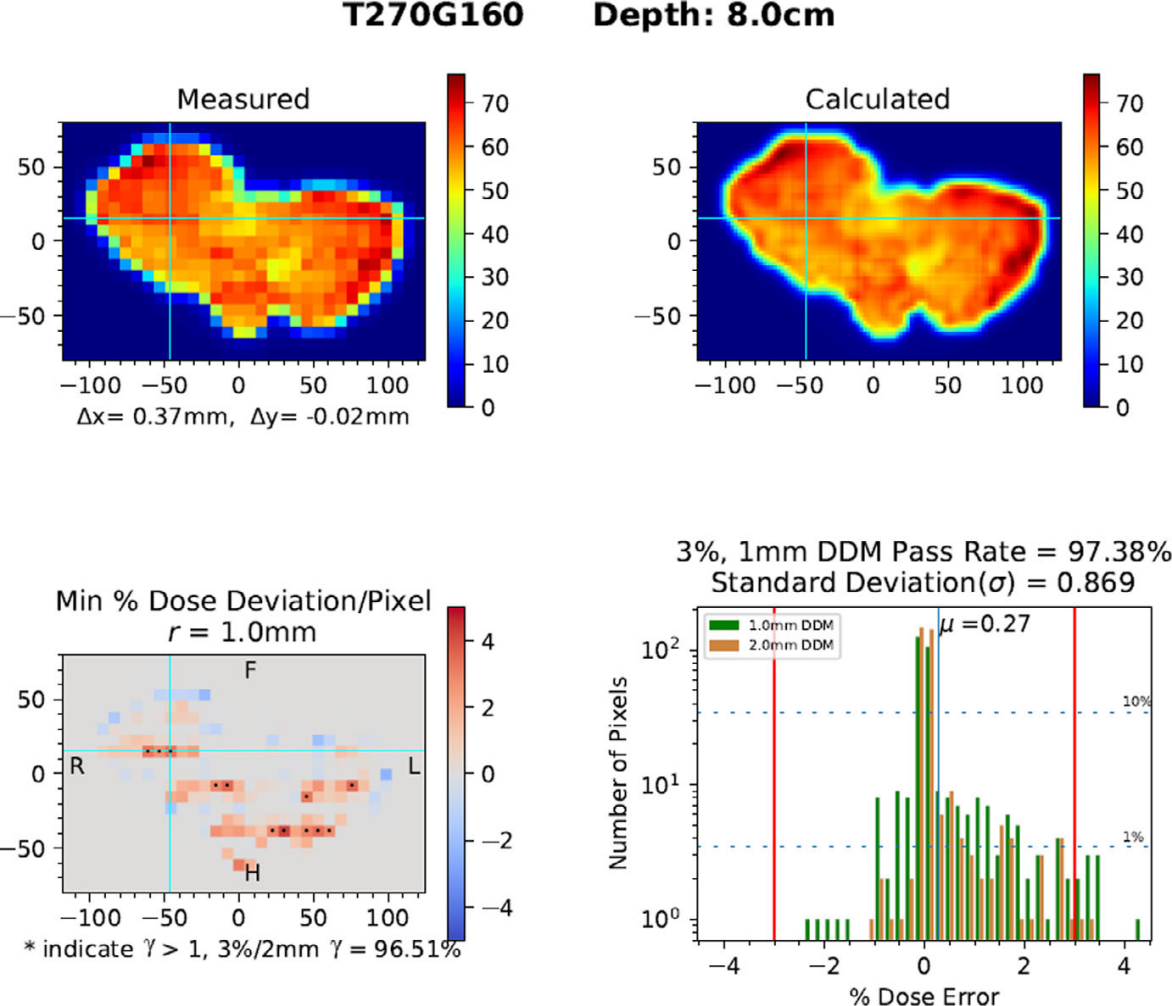
[Top row] Single plane dose measurement (left) and corresponding plane from calculated dose volume (right), image registration shifts are reported with Δx and Δy. [Bottom row] DDM heatmap (left), with distance hashes in mm, showing relative dose deviation in each voxel (Measured – Calculated), with voxels failing 3%/2 mm γ test marked by an asterisk; histogram (right) reporting magnitude and frequency of per‐voxel deviations along with DDM score, Standard Deviation (σ), and Average Dose Offset (µ).

The top row of Fig. 3 is provided for the reader to reference the measured and calculated dose planes used for comparison; the x and y represent image registration shifts used to align the fields. Registration was performed to provide a consistent comparison, isolate spatial deviations to the beamlets, and also to functionally track systematic shifts, such as laser offset. The bottom row of Fig. 3 provides additional information from a heat map, with astrisks overlaying the heat map representing failing pixels from a binary γ test. Due to the non‐elliptical area, *r* can be smaller than DTA for a given specificity (based on the AUC value, see Fig. 2), or the ability to convey reasonable similarity between planes, but the pattern of failing pixels for a 3%/1mm DDM still resembles that of the 3%/2 mm γ test, indicatd by the congruence of asterisks with out of tolerance dose scale on the heat map in Fig. 3. The passing rates for DDM (97.38%) vs γ test (96.51%) are also reported on this bottom row. The histogram can be used to readily quantify the frequency and magnitude of dose deviations as well as determine the average dose offset (µ), standard deviation (σ), and visual trends in the data for both a 1.0 mm (green) and 2.0 mm (tan) search distance. For illustration of the anlysis errors that may appear with a overly large search distance, the histogram for *r* = 2.0 mm was plotted alongside the 1.0 mm histogram in Fig. 3. This figure demonstrates two problems with an overly large search distance: 1. Allowing for false positives, that is, typically over reporting pixel counts at small dose deviations within histogram, indicated wherever the tan line exceeds the green; and 2. Under reporting pixel count at larger dose deviations, that is, tan lines tend to be less than green lines as histograms displays increasing per‐pixel dose deviation.

Red lines in the histogram represent the dose deviation tolerance, set at 3% in our clinic, and help to guide the user to quantify the maximum dose deviation (4.5%) for the few failing pixels. The y‐axis of the histogram is logarithmic in order to permit analysis of single pixels and deemphasize the strong majority of measurements with negligible dose disagreement; 1% and 10% dashed horizontal lines cross the plot to more intuitively show the percentage of pixels occupying each dose bin.

**Fig. 4:**
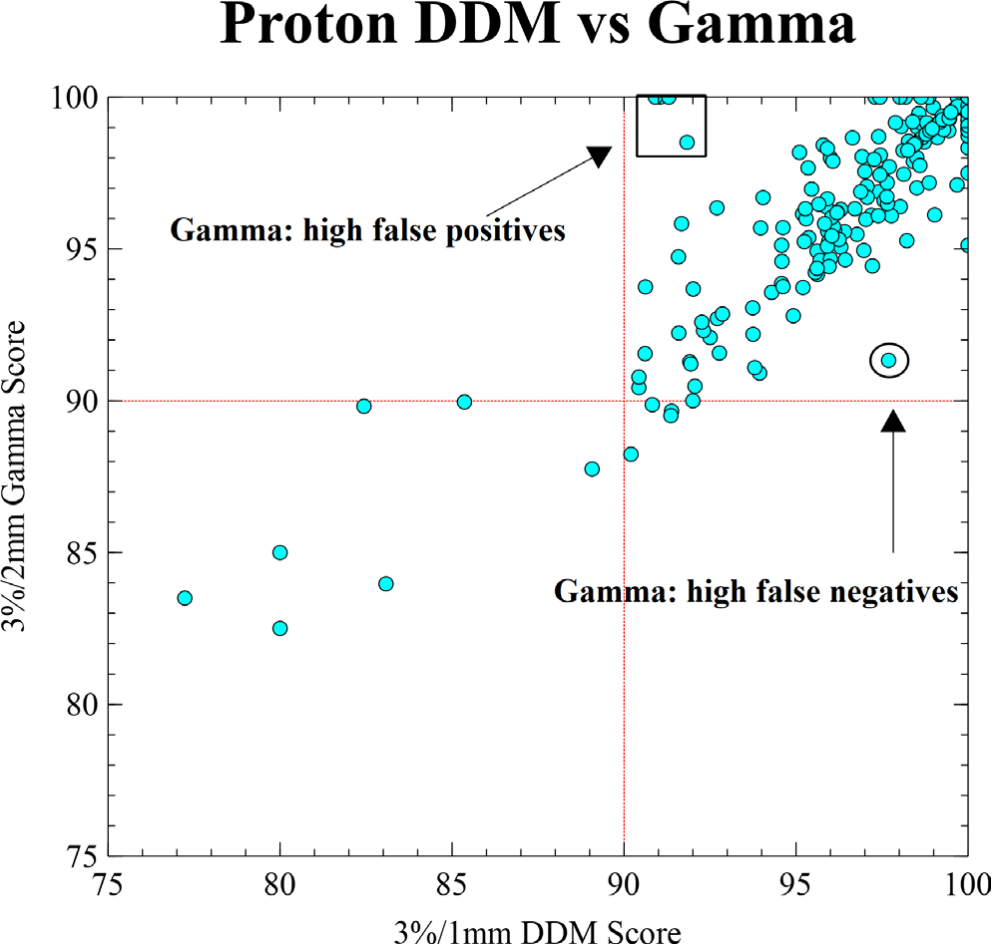
Comparison of passing rates between DDM technique and γ test for 75 proton plans (300 dose planes) measured on an IBA Matrixx. Outliers within rectangle represent relatively low DDM pass rates with atypically high γ pass rates driven by false positives; encircled measurements represent high DDM pass rates with relatively low γ pass rates driven by false negatives.

The passing‐rate agreement of the 3D DDM (90% pixels with a ΔD_min_(*r* = 1 mm) <3%, µ < 1%) with the 3%/2mm γ analysis for 75 IMPT patients (300 planar measurements, 1‐5 fields per patient) is displayed in Fig. 4. The mean passing rate for this comparison saw no change between a 3%/2 mm γ test (97.7 +/‐ 3.2%) and a 3%/1 mm ΔD_min_(*r*) (97.6 +/‐ 3.2%), largely because the γ test search pixels between 1 mm and 2 mm are irrelevant due to the overwhelmingly high probability of a proton spot to fall within a 1‐mm deviation.

**Fig. 5:**
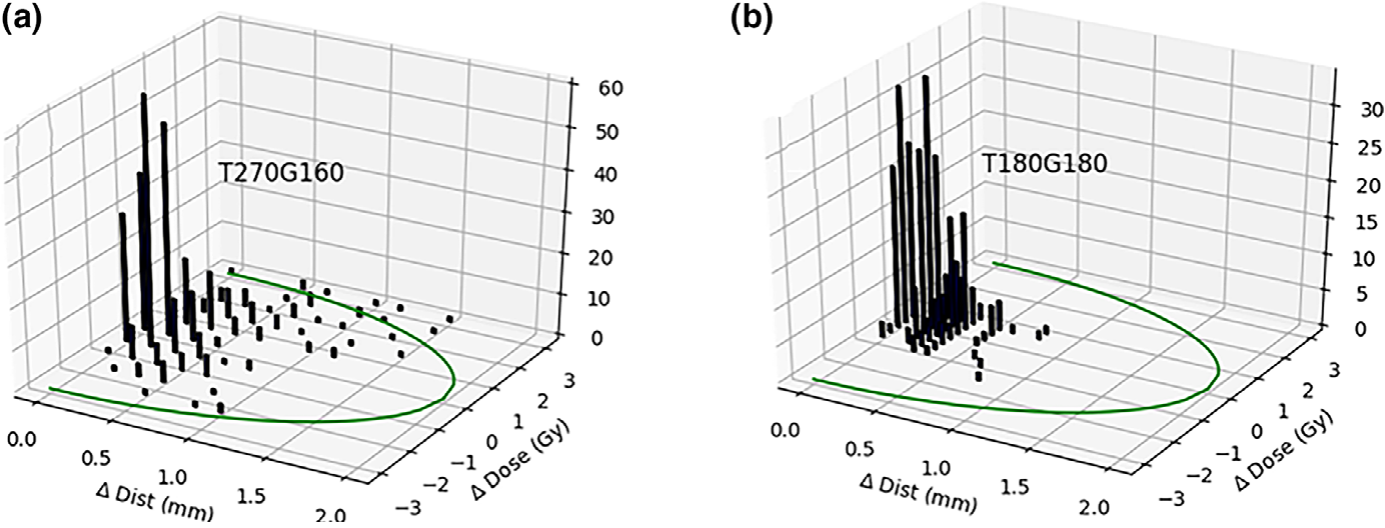
Histograms of each pixel’s minimum γ index from representative proton dose planes. The z‐axis represents number of pixels with minimum γ indices having similar dose and distance combinations. A 3%/2mm γ ellipse is marked in the X‐Y plane. Embedded graph titles are indicative of table and gantry positions during delivery.

The minimum γ index for each pixel was computed for 10 patients; Figure 5 shows histograms from two representative fields. An average of 97.8% (σ = 4.5%) of the γ indices were found within 1 mm of the correlated location.

## 4 DISCUSSION

This paper has presented a technique for dose distribution comparison. The method accepts the same inputs as the conventional γ test, and so may be used alternatively to or supplementarily with existing γ‐analysis workflows.

The proposed method carries forward pixel‐by‐pixel dose information that is masked by the dose and distance conflation inherent in the γ test. The newly proposed DDM method allows dose deviations to be read out on a continuum and analyzed potentially more intuitively, and can be run in parallel with the γ test for historical tracking as both tests operate using the same input data. The per‐pixel dose variation presented in the histogram is a key element of the proposed method. This histogram enables both statistical and enhanced intuitive evaluations which are not available in the γ test.

The statistical similarity observed between the two methods suggests that PSQA passing rates will not drastically change for institutions currently using a 3%/2mm γ test. However, the hope is that having more data available for analysis, presented in an intuitive, straightforward manner will improve the users ability to diagnose delivery errors and reduce the potential for errors to go unnoticed.

### 4.1 Sensitivity

The γ test has been rigorously analyzed and inadequacies have been noted in the literature, specifically with spatial insensitivity,^6^, ^9^, ^10^, ^11^, ^12^ and dose insensitivity.^5^, ^8^, ^10^, ^13^, ^14^, ^15^ Our objective is to optimize the AUC (Fig. 2) dimension by assessing only relevant distances and dose deviations. Spatial sensitivity is increased by eliminating the false positive results found in areas beyond the machine’s measured accuracy (*r*). The DDM analysis also addresses dose insensitivity by displaying a dose continuum so that slight deviations are observable, but only significant dose errors are penalized.

**Fig. 6:**
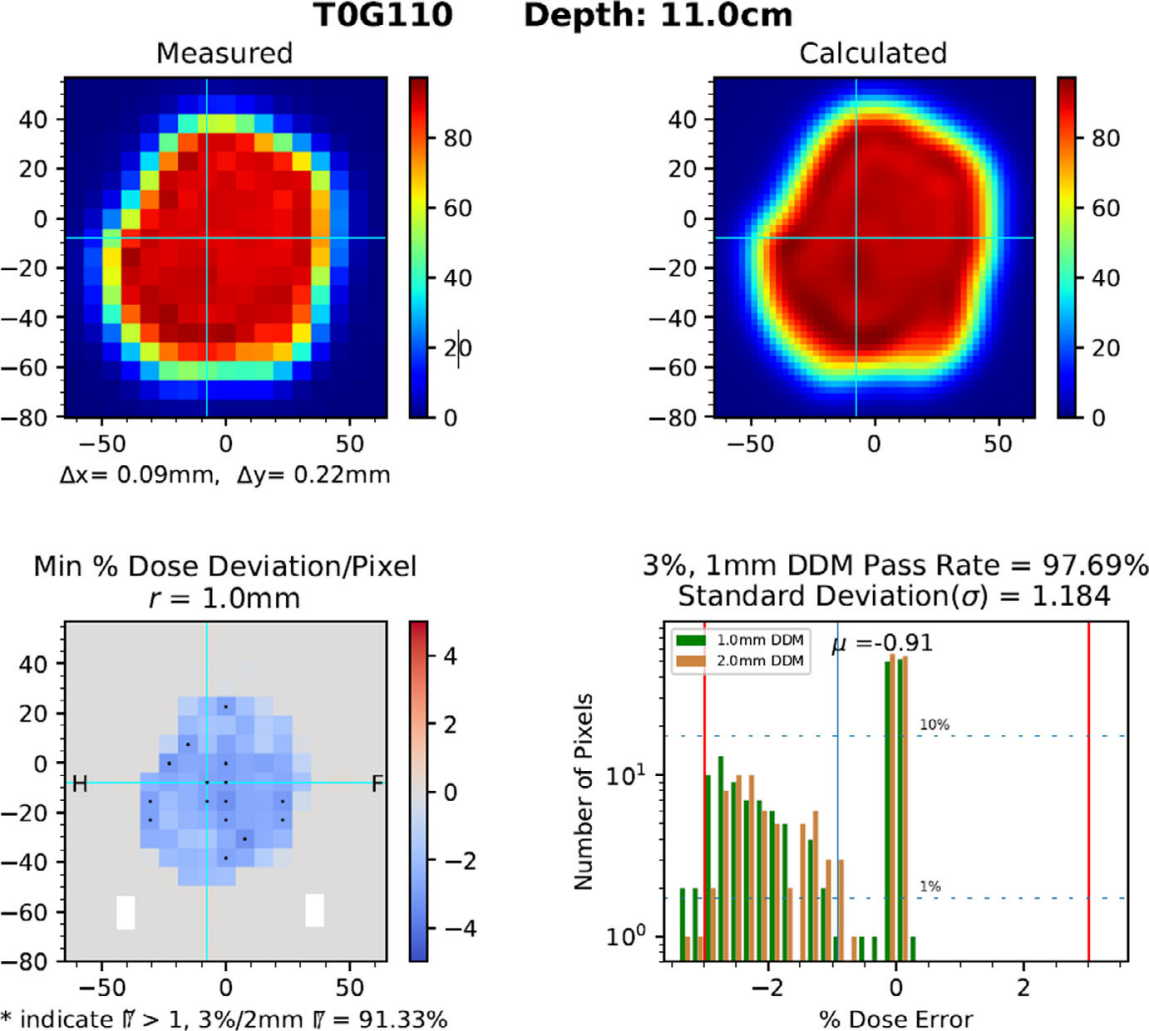
DDM output for a plan which received a uniquely large discrepancy between the γ test (91.3%) and DDM test (97.7%). The DDM per‐pixel tracking of deviation magnitude, demonstrates a global reduction in measured vs calculated dose.

Although the plot in Fig. 4 follows a fairly consistent trend indicating similar sensitivity between the two methods, some outliers exist. Data points corresponding to relatively high γ scores and low DDM scores are indicative of false positive γ‐test pixels that have passed with DTA values outside of the machine’s 3σ spatial accuracy. In contrast, outliers with relatively low γ scores and high DDM scores are likely indicative of a systematically high or low dose; one example of this is presented in Fig. 6. The DDM analysis indicates that the plan is consistently underdosed by nearly 3% with only the steep gradients passing. The γ equation would not convey the magnitude of a 3% dose offset but would produce a low passing rate due to the fact that a 3%‐dose discrepancy, which is widely considered to be within clinical tolerances, is mostly outside of the γ ellipse. The cylindrical search space of DDM, as well as carrying forward the dose difference per pixel in a straightforward manner, demonstrate that this plan’s deviations are within acceptable limits.

It has been observed that PSQA processes should correlate better to gold standards of field agreement.^15^, ^16^ The area inscribed in Fig. 2 indicates an increased sensitivity with this test as compared to the γ test. The analytical determination of the 1‐mm search distance used along with the observation that pixel failures in high‐dose‐gradient areas are extremely rare, evidenced by all measurements taken and all figures presented, can provide confidence that increased sensitivity is not a result of failing edges. The majority of gold standard plan delivery failures are due to dose deviation rather than distance deviation,^16^ and thus a more spatially accurate technique with more informative dose evaluation may bring PSQA into agreement with gold standards.

This work employed a tracked image registration as an essential step in shrinking the search parameter, *r*, to 1 mm to align with TG‐224 recommendations for beam and x‐ray isocenter deviation. Employing a proton‐capable PSQA detector with fiducials enabling x‐ray alignment would improve the method; discussions with vendors to this end have been promising.

### 4.2 Specificity

In Fig. 4, data clustering about the 45º line suggests that the specificity of a 3%/1mm DDM test is comparable to a 3%/2 mm γ test. Specificity of the DDM method is made more robust by generating search parameters based on more fundamental machine‐performance metrics.

Specificity is preserved with this technique by measuring beamlet uncertainties, tracking image shifts during registration, and ensuring the analysis of every statistically relevant test point for each measurement. In contrast, a 1‐mm γ‐test DTA includes less than 80% of relevant points for a selected dose agreement threshold (Fig. 2). Expanding the DTA to 2 mm allows users to capture just over 90% of relevant points, but doing so with an elliptical shape simultaneously encompasses numerous, irrelevant points outside of machine performance limits. Tracking the γ‐index DTA for 10 patients showed that about 98% of passing points fell within 1 mm (Fig. 5). The DDM‐proposed shape preserves specificity without adding irrelevant search points, demonstrated from the percentage of plans passed being similar to that achieved by others ^5^, ^17^ and to our own comparison with γ‐test results (Fig. 5). The histogram produced enables simple and accurate determination of maximum error and dose shift trends present in the measurement; this heightened access to data ensures that either large point‐dose errors or small dose shifts on large volumes will be recognized and appropriately evaluated by a responsible physicist. Neither of these potentially concerning scenarios are discernible with standard Boolean γ‐test procedures.

## 5 CONCLUSIONS

Field‐delivery spatial accuracy was well within 1 mm consistent with TG‐224 recommendations,^18^ as well as extensive QA and delivery logs suggesting that pixel‐comparison agreement outside of 1 mm is highly unlikely to occur, and is likely a false positive when it does. However, the elliptical shape of the γ test is too dose restrictive with a spatial error threshold set at 1 mm. The cylindrical search shape of the new DDM algorithm, proposed herein as more relevant to the quality of beam delivery, accepts all pixels within a given dose threshold inside the search radius. In addition, using a fixed dose threshold within an empirically defined search radius allows DDM to present the magnitude and direction of per‐pixel dose deviations in a straightforward manner to the user.

## CONFLICT OF INTEREST

The authors have no relevant conflicts of interest to disclose.

## AUTHOR CONTRIBUTIONS

Bryce Allred performed principle data collection, analysis and manuscript composition and was a co‐creator of the method described in the manuscript. Jie Shan and Wei Liu authored essential portions of the method code, and contributed to manuscript composition. Todd A. DeWees provided statistical support and analysis for the duration of the project, and composed portions of the manuscript. Jiajian Shen and Daniel Robertson collected data supportive to the method, and contributed to manuscript composition. Joshua Stoker oversaw the efforts of all individuals on the project, finalized the manuscript and was also co‐creator of the proposed method.
